# Data from Phase2/3 Clinical Study in Mild to Moderate Alzheimer’s Disease

**DOI:** 10.1002/alz.095125

**Published:** 2025-01-09

**Authors:** Cheng Fang, Melissa Gaines, Eve Damiano, Jenny Han, David Feng, Hilda Maibach, Maria L. Maccecchini

**Affiliations:** ^1^ Annovis Bio, Malvern, PA USA; ^2^ Wuxi/PharmaPace, San Diego, CA USA; ^3^ TCM, berkley heights, NJ USA; ^4^ IndigoRDD, Bethesda, MD USA

## Abstract

**Background:**

By inhibiting the translation of neurotoxic aggregating proteins ‐ amyloid precursor protein, tau, alpha‐synuclein etc., buntanetap restores axonal transport, lowers inflammation, and protects nerve cells from dying. Our Phase2 studies showed that buntanetap improved early AD patients ADAS‐Cog11 after a month treatment. We conducted a phase 2/3 study of buntanetap in over 300 mild to moderate AD patients (MMSE14‐24) to verify the findings.

**Method:**

Double‐blind, placebo‐controlled dose‐ranging phase 2/3 study to test buntanetap’s efficacy and safety in mild to moderate AD patients.

**Result:**

Patients were dosed with placebo, 7.5mg, 15mg and 30 mg QD buntanetap. A total of 353 patients were enrolled, and 325 patients completed the study across 54 sites in the US. Primary endpoints are ADAS‐Cog11 and ADCS‐CGIC.

Consistent with what we have observed before, buntanetap is very safe and well‐tolerated. Comparable numbers of adverse events (AEs) between treatment and placebo groups were observed. No serious AEs were related to buntanetap.

During the past year, commercial plamsa biomarker has advanced to the point that is comparable or superior to CSF biomarker and PET scan. We decided to use plasma biomarker to confirm the Alzheimer diagnosis. 202 patients were biomarker positive, identified as pTau217/npTau>4.2%. For the biomarker positive AD patients, 112 were moderate AD (MMSE 14‐20) and 90 were mild AD (MMSE21‐24). We found that buntanetap statistically significantly improved ADAS‐Cog11 in mild AD patients. After 3 months of treatment, all three buntanetap treatment groups showed statistically significantly improvement from their corresponding baseline (7.5mg improved 2.19 (0.87), p = 0.013; 15mg improved 2.79 (0.81), p = 0.001; 30mg improved 3.32 (0.82), P<0.001). Both 15mg and 30mg groups also had a statistically significantly improvement relative to placebo group. We didn’t observe a statistically significant difference in CGIC.

In accordance with the mechanism of action of buntanetap, we observed a reduction in plasma np‐Tau (non‐phosphorylated Tau) after treatment, providing further credence to buntanetap’s efficacy and mechanism of action.

**Conclusion:**

This short study shows a symptomatic effect with a possible disease‐modification trend according to the tau data. The next study will have a longer duration, improved design and be statistically powered to validate symptomatic improvement and disease‐modification.

